# Return of research results (RoRR) to the healthy CHRIS cohort: designing a policy with the participants

**DOI:** 10.1007/s12687-021-00536-1

**Published:** 2021-07-09

**Authors:** Ciara Staunton, Maria Kösters, Peter P. Pramstaller, Deborah Mascalzoni

**Affiliations:** 1 Institute for Biomedicine, Eurac Research, Affiliated Institute of the University of Lübeck, Via Galvani 31, 39100 Bolzano, Italy; 2 School of Law, Middlesex University, Room WG35, The Burroughs, Hendon, London, NW4 4BT UK; 3 Department of Neurology, Central Hospital, 39100 Bolzano, Italy; 4 Department of Public Health and Caring Science, Uppsala University, CRB, P.O. Box 256, 751 05 Uppsala, Sweden

**Keywords:** Unsolicited findings, Return of genetic research results, Genetic research, Research policy, Healthy cohort study

## Abstract

Legal, financial and organizational challenges and the absence of coherent international guidelines and legal frameworks still discourage many genetic studies to share individual research results with their participants. Studies and institutions deciding to return genetic results will need to design their own study-specific return policy after due consideration of the ethical responsibilities. The Cooperative Health Research in South Tyrol (CHRIS) study, a healthy cohort study, did not foresee the return of individual genomic results during its baseline phase. However, as it was expected that the follow-up phase would generate an increasing amount of reliable genetic results, an update of the return of research results (RoRR) policy became necessary. To inform this revision, an empirical study using mixed methods was developed to investigate the views of CHRIS research participants (20), local general practitioners (3) and the local genetic counselling service (1). During the interviews, three different examples of potential genetic results with a very diverse potential impact on participants were presented: breast cancer, Parkinson disease and Huntington disease. The CHRIS participants also completed a short questionnaire, collecting personal information and asking for a self-evaluation of their knowledge about genetics. This study made it clear that research participants want to make autonomous decisions on the disclosure or non-disclosure of their results. While the motivations for participants’ decisions were very diverse, we were able to identify several common criteria that had a strong influence on their choices. Providing information on these factors is crucial to enable participants to make truly informed decisions.

## Introduction: background

Technical advancements and decreasing costs of exome and genome sequencing have led to their rapidly expanding application in research and thus to the generation of an increasing amount of reliable genetic results. Within the scientific community, consensus is growing that individual research results, which are analytically valid and clinically actionable, should be shared with research participants (Knoppers et al. 2015; Budin-Ljosne et al. 2016; Mackley et al. 2017). Those results could come as unsolicited results that might arise during further research, and might also be communicated to participants (Ewuoso 2016; Souzeau et al. 2016; Mackley et al. 2017) on the basis that it raises the possibility of enabling appropriate medical treatment and possibly preventing serious illness (Steinsbekk and Solberg 2012; Christenhusz et al. 2013). Moving beyond a clinical justification, sharing results shows appreciation for participants’ contribution (McGuire and Lupski 2010), can strengthen a trustful relationship between researchers and participants and could lead to an increase in willingness to participate in further research studies (Kaufman et al. 2008).

Importantly, empirical evidence demonstrates that most research participants not only want to have access to their results (Bollinger et al. 2012; Facio et al. 2013; Allen et al. 2014; Middleton et al. 2016; Jamal et al. 2017; Yamamoto et al. 2017, 2018), they might even feel deceived or betrayed if results are withheld (Dresser 2014). Based on these findings, in order to respect participants’ autonomy, researchers must at least offer the results to research participants. However, this desire or right to know must be balanced with participants’ “right not to know”. Offering results must be done in a manner that respects this right not to know and desire not to be told about the genetic information even if it is clinically actionable (Andorno 2004; Domaradzki 2015; Hofmann 2016). To truly respect the autonomy of the participant in this domain, the final decision on actual disclosure must always remain with the participant, who should have the possibility to decide what kind of results they want to receive (Angrist 2011; Budin-Ljøsne 2012).

There are, however, a number of challenges in the feedback of results. First, within the scientific community, there is a lack of consensus on the results to be returned. Some estimates locate the potential number of genetic variants meeting the threshold for disclosure today between 11,000 and 15,000 (Cassa et al. 2012). The American College of Medical Genetics (ACMG), however, recommends only 59 medically actionable gene variants for return (ACMG Board of Directors 2015) and assigns the researchers an obligation to actively screen for and report pathogenic variants (Mackley et al. 2017). The European Society of Human Genetics (ESHG) endorses a more cautious approach, recommending the targeted sequencing and analysis of genetic data in line with the primary research goals (Matthijs et al. 2016; Mackley et al. 2017), in order to keep the number of unsolicited results as low as possible (van El et al. 2013).

Second, it is not always clear who should have the responsibility to feedback the results, particularly in the context of data sharing and biobanks. There is a lack of consensus as to whether it should be the original researcher, the biobanks providing the data, or the current researcher who has responsibility for returning unsolicited results created by others (Bledsoe et al. 2013).

Third, there are concerns as to the potential liability of researchers. In cases of non-disclosure, this may be as a result of failure to disclose results that potentially could have prevented harm (McGuire et al. 2014). In cases of disclosure, this may arise due to the harm arising from disclosure of distressing genetic results.

Outside of the possibility legal responsibilities and liabilities of researchers, learning their carrier status of a genetic variation and the possibility of developing a disease may have severe negative psychological and social consequences for participants (Bredenoord et al. 2011). Therefore, it is essential that participants are informed about the possible implications of their decisions to be fed back genetic results. Providing comprehensive and easily understandable information is critical to enable truly informed decisions by participants on this matter. An additional layer of complexity is that the unsolicited results may relate not only to the research participant, but also their family members who may be potential carriers of the genetic mutation (Wolf et al. 2015).

In light of these concerns, in order to return any results, a comprehensive strategy must be put in place allowing a responsible disclosure process. This will impact the informed consent process, have implications on the management of re-contact of participants, require clinical validation of research results to be conducted, and necessitate the provisions of genetic counselling (Budin-Ljosne et al. 2016).

To meet these demands, additional resources will need to be allocated, a requirement many research funders may be unwilling or unable to finance (Budin-Ljosne et al. 2016). Costs of disclosure and who holds the burden of return are a serious issue to take into consideration when discussing the RoRR. In fact, the responsibility for allocating additional resources could fall to the local healthcare systems. In recent years, an increasing number of national healthcare organizations have invested in large-scale genome sequencing projects. Stark et al. (2019) and Manolio et al. (2015) map an extensive list of publicly funded genomic-medicine initiatives around the globe and some provide genetic counselling to their participants. Despite this, it is still questionable whether national healthcare services *should*, are *willing*, and are *able* to take over the communication of genetic research results to research participants. Issues such as the lack of infrastructure to process the genetic data, limited capacity and capability of clinical staff to interpret genetic results, uncertain clinical utility and validity of genetic results, and extensive additional costs will need to be overcome (Stark et al. 2019; Manolio et al. 2013). Thus, Ginsburg (2014) argues that the majority of healthcare systems around the world are not ready yet to translate results of genetic research into practical healthcare.

In light of these legal, ethical, financial and organizational challenges with communicating genetic results, it is unsurprising that policy makers are grappling with the development of policies and best practice (Knoppers et al. 2015; Fiallos et al. 2017; Budin-Ljosne et al. 2016). In the absence of guidelines, most studies still decide not to return any individual research results (Heaney et al. 2010). Studies that opt to feedback genetic results will thus need to design their own study-specific return policy after due consideration of the aforementioned ethical and legal responsibilities. A patient-centered approach guided by participatory research such as community-based participatory research (CBPR) that focuses on empowering participants and giving them an active role in developing research processes should be adopted (Institute of Medicine 2013; Wells and Jones 2009; Chung et al. 2010; Anderson et al. 2012; Kost et al. 2017; Vayena and Tasioulas 2015). This is particularly important in developing a returns policy where new sequencing techniques may unveil a great number of relevant variants in healthy cohorts.

### The CHRIS study

The Cooperative Health Research in South Tyrol (CHRIS) study is a healthy cohort study that explores the genetic and molecular foundation of cardiovascular, metabolic, neurological and psychiatric diseases in the general population of the middle and upper Vinschgau/Val Venosta in South Tyrol, Italy. The CHRIS study is a population-based study with a longitudinal outlook, which started in 2011 (Pattaro et al. 2015). The baseline study phase was completed in December 2018 with over 13,000 participants. The follow-up phase, in which all participants will be re-invited, started in 2020. Until now, the quality of the genotyped data derived from the CHRIS study did not allow the clinical validation of any genetic results. Therefore, the policy of the baseline study regarding genetic results neither foresaw an active search for known diagnostic loci nor foresaw a general return of individual genomic results to research participants. This was communicated to participants before and during the CHRIS study. From the beginning, it was explained that the return of individual genetic results was generally not foreseen and that the researchers of the CHRIS study are not actively looking for diagnostic variants. It was also specified that the research of the CHRIS study is experimental and that it is highly unlikely that relevant individual genetic results will be found. This was communicated in a participant brochure and a video as an integral part of the informed consent process. After watching the video, participants were asked to indicate their preference on re-contact if, in exceptional cases, unsolicited results were discovered. Participants could choose between one of 4 options: (1) to be contacted if any results were discovered; (2) to be contacted only if treatment options are available; (3) to be contacted if family members might be affected; or (4) never. During the baseline phase, a majority of 88% of participants opted for re-contact in any case and less than 1% for no re-contact at all.

To prepare for the cases of unsolicited results during the baseline study, the CHRIS study protocol adopted a strategy that established a committee consisting of the researchers involved in the analysis of the genetic data, the principal investigator of the specific study, members of the genetic counselling group from the local hospital of Bolzano, the President of the ethical review board and other members, depending on the trait. The committee would evaluate if the found results are clinically valid and actionable. Next, the participants’ consent form would be assessed to determine if they gave their permission to be contacted about individual genetic results. Following this, if the Committee decided that the results are to be returned, the genetic counselling service would invite the affected participants for an individual consultation and perform clinical sequencing to reassess the found genotype. Any costs for the counselling procedure and further tests or treatment would be met by the local healthcare system. Until now, no results have met the threshold to be discussed within the committee.

The return policy for the baseline phase of the CHRIS study was developed by Deborah Mascalzoni after consultation with the local genetic counselling service. The policy was innovative when it was formulated in 2011 as very few genetic studies included the return of any genetic results in their study policies. Since then, there have been considerable changes in ethical best practice. Furthermore, due to increasing research on CHRIS data and growing knowledge about the significance of genetic variants, we expect to be confronted with an increasing number of relevant individual genetic research results soon. A revision of the return policy was then considered to be necessary. As part of this revision, we sought to gain insight into the concerns, needs and wishes of CHRIS research participants regarding the return of individual genetic results. As the return strategy of the baseline phase foresaw that the local genetic counselling service would take over the necessary clinical validation and initial communication of the genetic results and the medical consultation, the insights of genetic counsellors were sought. Additionally, we also strove to understand the concerns of local general practitioners (GPs) and their perception of practical challenges. In South Tyrol, an area characterized by small villages and mountain agriculture, the population still has a strong trustful relationship with their local GP. It is expected that many participants receiving genetic results will communicate these results to their GP and seek their advice. Thus, it was necessary to assess how best to integrate them in the planning process, evaluate their needs and wishes regarding information and potentially education in the return of results. This paper reports on the findings of this research and will be used to inform the revision of the CHRIS returns policy.

## Methods

This was a qualitative research study involving face to face interviews with 20 CHRIS research participants (out of more than 13,000), 3 GPs (out of 16 working in the area involved) and the head of the only genetic councelling unit in south Tyrol. This unit is the one that would be involved in the RoRR if performed. During the baseline study, participants were asked if they would be willing to take part in an additional interview regarding the return of individual genetic results. Out of the volunteering participants, a purposive sample (Neuman 2011) was selected, ensuring gender-balance and covering different age groups. The interviews were continued until no new arguments concerning the return of results arose and were conducted on 5 different days between October 2017 and February 2018 with 11 female and 9 male participants. The youngest interviewee was 18, while the oldest was 76 years old.

This study was reviewed and approved by the “Comitato etico della provincia di Bolzano SABES” as part of the Cooperative Health Research in South Tyrol (CHRIS) study.

### Data collection

#### Interviews with CHRIS participants

During the baseline study, a general written consent was obtained for the participation in the CHRIS study comprising the conduction of interviews. In addition, participants were asked for their verbal consent to audio record the interview.

Prior to starting the interviews, participants were invited to complete a short questionnaire, collecting personal information and asking for a self-evaluation of their knowledge about genetics. Additionally, the questionnaire comprised 9 statements on inheritance and the development of genetic diseases, which had to be rated true or false. These, in addition to the self-evaluation questions, were included to assess participants’ level of genetic education as it might correlate with their opinion on receiving genetic results.

During the interviews, participants were presented with three examples of hypothetical individual genetic results that could potentially emerge through the CHRIS study or follow-up studies: breast cancer, Parkinson disease, and Huntington disease. The examples were carefully selected, based on a literature review and under consultation with geneticists at the Institute of Biomedicine, Eurac, in order to represent three different typologies to better illustrate the great diversity of genetic results regarding their informative value on health/life implications, their medical utility, and actionability. The examples showcase the differences of genetic variants in several characteristics, namely, the risk of developing the disease due to the genetic mutation, the availability of preventive measures and medical treatment options, the curability and potential lethality of a disease, and the age of onset of the disease. Table 1 presents the different expressions of characteristics for each genetic variant. Table 2 contains the translated information provided to the interview participants about the nature of genetic results and about each potential genetic finding and an introduction given. Participants were encouraged to ask questions in order to help them properly understand the different examples.

**Table 1:** Characteristics of main examples of genetic results

	Breast cancer	Parkinson disease	Huntington disease
Genetic mutation	Autosomal, dominant mutation BRCA1 and BRCA2	Low penetrance, recessive, heterozygous mutations in the parkin gene (PRKN)	Autosomal, dominant mutation in the Huntingtin gene (htt). Monogenic disorder
Risk (of developing the disease due to the mutation)	Significantly increased risk of 38–87%	Slightly increased risk (3–7%)	High risk (100%)
Prevention possibilities	• Regularly precautionary medical check-ups • Precautionary mastectomy • Early recognition significantly increases the chances of complete recovery • Late recognition, in which metastases have formed in other body regions prohibits complete recovery	No preventive measures available	No preventive measures available
Treatment possibilities	• Surgery • Chemotherapy • Radiation • Hormone therapy	• Treatable symptoms. Measures to increase life quality	• Medication • Physiotherapy • Occupational therapy • Speech therapy
Disease onset	Earlier than without mutation	Adult onset at an average age of 60 years	Adult onset between 40 and 50 years
Cure	Sometimes possible	No cure	No cure. Premature death
Severity of the disease	Potentially lethal	Non-lethal	Lethal (premature death after 15–18 years after onset)

**Table 2:** Information provided orally to participants on examples of potential genetic results (translated from German)

Example	Description
General introduction (given directly before presentation of examples)	“At this point in time you will only receive clinical results from the CHRIS study, which are among others your ECG, blood results and your Body-Mass-Index. This however might change in the future. With advancing research, we expect that more individual genetic results will arise, that might be relevant for our participants. Therefore, we are trying to figure out what kind of genetic results might be interesting to our participants. And this is also why we would like to know more details from you on what kind of genetic results you would like to receive Generally, the genetic research of the CHIRS study is still experimental and thus we do not expect to detect many results, that have a direct medical use for a single research participant. However, with increasing research on the collected data it might be the case, that now or in the future, results are found, that are indeed relevant for the health of our participants It is important to know that genetic results are can be very diverse. Only a very small number of results can predict if someone will develop a disease with high certainty. A much larger number of results will only be able to indicate an elevated risk to develop a certain illness To make it easier for you to understand what genetic results are, I will present you with three examples of potential genetic results. These examples are very different and are representing different types of genetic results. After each example, I will ask you what you think of the result and if you would like to receive it. If you have any question during my explanation, please do not hesitate to interrupt me I want to stress: These examples are presented in a simplified manner and we are not going to much into scientific details. Furthermore, the decision you are making today is not binding for any actual genetic results from the CHRIS study Do you have any questions so far?”
BRCA1/BRCA2	The first example are two genetic variants, that increase the risk of developing breast cancer. Approximately 60 to 80 out of 100 women, that have one of these genetic variants, will develop breast cancer at some point throughout their life. Not only women also men can develop breast cancer due to these variants. However, they have a lower risk than women. *(In men it is only 20 out of 100). The risk of developing breast cancer due to these genetic variants is relatively high, however it is not 100 percent. This means, that a person that has this genetic variant might stay healthy throughout their entire life. Moreover, also a person, that does not have the genetic variant, might still get breast cancer If one of these genetic variants has been detected, it is possible to do regular precautionary medical check-ups. This will facilitate the recognition and treatment of a tumor at an early stage and increase the chances of a complete recovery significantly. Another possibility is to have a precautionary breast removal on both sides. *(As a man, you might not feel too concerned about breast cancer. However, keep in mind that breast cancer is only an example representing similar kinds of results. Thereby I mean genetic variants that lead to a high risk of developing a serious disease for which precautionary measures are available.) The chances to pass the genetic variant on to children are 50 percent. This does not mean that children will develop the disease, but that they can inherit the elevated risk of developing breast cancer Do you have any questions about this example? *Information in brackets has only been provided to men
Morbus Parkinson	The second example is a genetic variant, that increases a person’s risk of developing Parkinson Disease. Have you heard of the Parkinson’s Disease before? (pause for answer) Parkinson is a disease of the central nervous system. One of its best-known symptoms is heavy tremor. Other symptoms are muscle rigidity and cognitive impairments So far, several genetic variants have been identified, that increase the risk of a person to develop Parkinson Disease. However, the augmentation of the risk is very low. This means the chance of actually developing Parkinson Disease because of one of those genetic variants is very low. Actually, the risk is only slightly higher than of any other person of the population to develop Parkinson Disease. Moreover, most people that develop Parkinson Disease, do not have any of the known genetic variants In contrast to breast cancer there are no precautionary measures that can be taken, to prevent the development of Parkinson Disease. The outbreak of the disease cannot be prevented, and treatment is only possible once the disease manifested itself. Until now Parkinson Disease cannot be cured. However medical treatment can alleviate the symptoms significantly, and the progress of the disease can be delayed. Also, it is important to know that Parkinson Disease does not necessarily lead to a premature death The chance to pass the genetic variant on to children is 50 percent. Like for breast cancer, this does not mean that children will inherit the disease, but that they might inherit an elevated risk to develop it Do you have any questions about this example?
Chorea Huntington	A very different result is a genetic variant that causes the Huntington Disease. Have you ever heard of this disease? (pause for answer) Huntington Disease is a disease that is caused by a single genetic variant. It damages the neural cells in the brain, which leads to the development of motoric and psychological problems like involuntary movements, alterations of the personality, depression, dementia and psychosis. In the final stage of the disease the affected persons are confined to their beds and completely dependent on the help of others. The disease usually sets on around the age of 45. The disease is not curable and always leads to a premature death The genetic variant causing the disease can be detected through a genetic test. A person that has this genetic variant will always develop the disease. Moreover, the genetic variant can be passed on to one’s children. The chances for children to inherit the variant is 50 percent. If a child has inherited the variant it will develop the disease at one point in their life Do you have any questions about this example?

In case participants asked for more or different examples of potential genetic results, two additional examples had been prepared: malignant hyperthermia (MH) and restless legs syndrome (RLS) (Tables 3 and 4). These examples were chosen because they highlight additional characteristics of genetic results. Potentially lethal complications during medical interventions under anesthesia cause MH that can be entirely prevented by using alternative anesthesia if the genetic predisposition is known. This high benefit of knowing one’s carrier status paired with the low chance of detecting it without genetic testing made MH a good example for those participants skeptical of receiving any genetic results. RLS was chosen because in contrast to the other examples of potentially very severe diseases, it represents a non-lethal illness for which no preventative measures exist. Additionally, the symptoms of the disease can be strongly improved symptoms once they occur. The additional examples were also prepared in case a participant had personal experience with one of the main examples and their decision thereby biased and not based on the general characteristics of the genetic result. During all interviews, two researchers were present (M.K. and D.M). This approach was chosen, as it proved useful for one interviewer to ask the questions and for the other to follow the conversation and add additional queries when statements seemed to be unclear or ambiguous.

**Table 3:** Characteristics of alternative examples of genetic results

	Malignant hyperthermia	Restless legs syndrome (RLS)/Willis-Ekbom disease
Genetic mutation	Autosomal, dominant mutation RyR1 or CACNA1S	Polygenic disorder. Various variants contribute to the phenotype
Risk (of developing the disease due to the mutation)	High risk (50%)	Slightly increased risk
Prevention possibilities	Usage of alternative anesthetic agents or muscle relaxants	No preventive measures
Treatment possibilities	If recognized promptly, treatable. Otherwise possibly lethal	Lifestyle change and medication can alleviate symptoms
Disease onset	Anytime	From childhood to over 90 years
Cure	No cure	No cure
Severity of the disease	Potentially lethal	Non-lethal

**Table 4:** Information provided orally to participants on alternative examples of potential genetic results (translated from German)

Example	Description
Malignant hyperthermia	Another example for a possible genetic result is Malignant Hyperthermia Malignant Hyperthermia is a condition that can lead to severe complications if certain anesthetics or muscle relaxants are given to a person, for example in the course of an operation. These complications can lead to organ failure and end in death. However, if the cause of the complication is identified correctly measures can be taken that to cure the symptoms and death In case it is known that a person has the genetic variant responsible for Malignant Hyperthermia, alternative anesthetic agents will be used, and the complications can be prevented altogether The chance to pass the genetic variant on to children is 50 percent. This does not mean that children will inherit the disease, but that they might inherit the risk of developing it Do you have any questions about this example?
Restless legs syndrome	A further example for a possible genetic result is the Restless Legs Syndrome. The disease leads to the development of an unbearable urge to move your legs or sometimes also arms during rest or night-time. This often results in insomnia and sleep deprivation and can have negative effects on the mental and physical health of a person Several genetic variants have been identified that increase the risk of developing this disease. However, not just genetic factors but also other factors like lifestyle or environmental factors can influence the development of the Restless Legs Syndrome At the moment, the Restless Legs Syndrome cannot be cured but lifestyle changes and medication can significantly ease the symptoms. The disease can develop at any age The chance to pass the genetic variant on to children lies between 50 and 60 percent. This does not mean that children will inherit the disease, but that they might inherit the risk of developing it Do you have any questions about this example?

The questions focused on four main topics: (1) the participants’ motivation for their participation at the CHRIS study; (2) their knowledge of genetic diseases; (3) their preferences and concerns regarding the return of individual genetic results; and (4) their wishes regarding practicalities of the returning process. These topics were developed based on literature review that lead to a first publication (Budin-Ljosne et al. 2016) and empirical research results from an unpublished study with local GPs (D.M.) identifying current challenges impeding the communication of genetic results to research participants. The interviews lasted between 20 and 40 min and were held in German, the main language spoken in the region.

#### Interviews with general practitioners and a genetic counsellor

The GPs and genetic counsellor interviews focused on the following themes: (1) expertise and experience in communicating genetic results in their everyday work; (2) opinions on returning genetic results to CHRIS participants; (3) opinions on an active search for diagnostic variants; (4) the potential role of GPs/genetic counselling services in the return process. The participants were also presented with the same three examples of potential genetic results and inquired on their opinion on communicating these results to research participants.

All interviews were conducted in German, except the one with the head of the genetic counselling service, which was conducted in Italian. After obtaining verbal consent, the interviews, which lasted between 30 min and 1 h, were audio recorded. One interview with a GP was conducted via telephone with only one researcher (M.K.), while all others were held face to face with two researchers (M.K. and D.M.).

### Data management and analysis

The interviews were audio recorded, transcribed verbatim, and coded thematically using Atlas.ti (M.K.). Both M.K. and D.M. independently coded the transcripts and any discrepancies were discussed. The coding was then revised as was the codebook (M.K.) (Neuman 2011; Flick 2014; Belotto, 2018). All analytical steps were performed in German, also for the Italian interview. Only the results of this study were translated into English for this paper.

## Results

After being asked if they wanted to be informed about a specific genetic variant, should they be found to be a carrier, 17 participants wanted to be alerted about the variants BRCA1 and BRCA2, while 3 declined. In the event of mutations in the parkin gene, 7 individuals expressed the desire to be notified, 10 decided against it, and 3 remained undecided. Nine participants asked for disclosure of the variant causing Huntington disease, while 11 did not wish to learn about it.

During two interviews, in which the participants did not want to be informed about any of the three presented genetic variants, the interviewers decided to present MH as an additional example. Both participants stated that they wanted to be notified if they had the variant related to MH.

In one interview, a participant stated that they did not want to be informed about variants related to Parkinson disease, as they had personal experience with the disease. The participant also did state that they would not want to be informed about any variant with potentially severe health implications, no matter the variant’s other characteristics. Following this, the interviewers decided to present the example of the restless legs syndrome (RLS), as it has similar characteristics to Parkinson disease (no, preventative measures, no cure) but with less severe health consequences. The participant wanted to be informed about RLS.

### Disclosure—an intrinsic decision

The choices made by participants and the motivations behind them proved to be extremely diverse and highly personal. A common observation, however, was that participants wanted to make an autonomous decision regarding the disclosure or non-disclosure of their results. Some stated that they would feel betrayed or disappointed if results were available but not offered to them. Several participants felt that they have a right to be informed about available personal genetic results, even if it might be categorized irrelevant by researchers or genetic specialists. Participants felt that decisions on disclosure of results must remain with the individual and that these preferences may change over time in accordance with their own preferences and changes in scientific knowledge.

Some participants allocated a clear responsibility to the study to inform and help study participants and therefore share results. However, some stated that the CHRIS study should serve the common good and therefore would understand if no results are offered due to economic limitations.

### Decision factors for disclosure

Although factors affecting decision were highly personal, there were some common criteria. They were actionability of results, disease factors, and impact on genetic relatives.

#### Actionability of results

The medical actionability of genetic results played a central role in participants’ decisions on disclosure. Participants were presented and discussed four subcategories of actionability:

The possibility to entirely prevent the onset of a disease;

The possibility to recover completely after the onset of a disease which cannot be prevented;

The availability of precautionary measures that cannot prevent the outbreak of a disease but facilitate early recognition and timely treatment and potentially cure;

The availability of medical treatment or lifestyle changes that cannot cure the disease but delay its progress or ease its symptoms.

Fear of experiencing anxiety was a cross-cutting argument against disclosure of results of all categories, while the key argument for disclosure was the possibility to take actions and the feeling of being prepared for the future. Participants weighed these factors against each other in their decisions and made very diverse choices.

##### Possibility to prevent onset of a disease

All participants wanted to be informed about results that could facilitate the complete prevention of the onset of an illness. These results were seen as a unique opportunity to take active measures to stay healthy and the probability to suffer from anxiety was evaluated as low.

##### Possibility to cure a disease

Genetic variants that increase the risk of developing a disease which can be cured once it breaks out, but not prevented beforehand, led to diverse decisions on disclosure. Participants deciding for disclosure reasoned that because the disease was curable, they would not worry, but be able to inform themselves early on about risks and treatment options.

[…] you will be more attentive, if something is out of the ordinary. That you rather say, now I will let this be checked or so… Or otherwise maybe… ahm you will not pay attention to it and then it might be too late. (Participant 02).

Other participants saw no advantage in knowing these genetic predispositions because they cannot take any proactive measures before any symptoms show. They feared burdening their lives with unnecessary anxiety as the disease might never break out, and if it did, they would still be able to undergo medical treatment and cure the disease.

No, I would not want that. As soon as it occurs, I will have to handle it, but I don’t want to know. […] Because maybe then in my fear…. I would live in fear it could be affected or I could get this disease and that I do not want because I still cannot do anything against it. (Participant 01).

##### Availability of precautionary measures to treat or cure

Where results would enable participants to take precautionary measures that cannot prevent the disorder but lead to early detection and treatment, a number of participants stated that knowing their results would motivate them to take precautionary measures or have regular medical check-ups. The possibility to act before a disease might break out as well as the fear of missing the chance of intervening early enough led participants to decide for disclosure.

[…] If you know that, you might be more diligent with the check-ups. Otherwise you always think: ahh not me, or, yes yes I will do that sometime, or … exactly and I think if you know you are in this thing, that it could be…, I think you will be more diligent with the check-ups. You will say, yes ok now I will do this once a year. (Participant 02).

Others, however, feared they would burden their lives with stressful medical visits that will not be able to prevent the development of the disease. These participants felt that concerns on the suffering of anxiety outweighed the benefit of the limited actions available.

I would not make the test, because if I get it, I get it (breast cancer), and I don’t care if I have a higher risk or not. […] I would not … how do you say … visit the doctor more frequently, because I have it (genetic variant). I would not do it. (Participant 9).

All participants expected that results for diseases that can be eased through medical interventions but not completely cured will cause worries. However, some regarded the possibility to mentally prepare themselves for the consequences of a disorder as more valuable. They expected that the availability of medical treatment options would ease their worries and led them to decide for disclosure. One participant also explicitly stated that if traditional medicine cannot provide a cure, they would search for treatment or prevention options from alternative medical schools like traditional Chinese medicine.

… there are maybe people that, let’s say, sleep away, their life. And I think if someone has something like this (Huntington disease), then he can prepare himself. Also things like arrange things at home, like the last will, because I think later you cannot do this anymore. And things like that. Yes, I think that is important (Participant 11).

Other participants felt that since no actions can be taken to prevent or cure the disease, knowing their genetic predisposition was pointless. They feared such results would cause anxiety and thus lower their life quality. Moreover, they also expressed the concern that knowing their results might alter important decisions in their lives.

The difference is, you cannot do anything against it, it does not matter if I know it sooner or later, it is here when it is here. And because you cannot do much, I do not think it is like breast cancer, that is something else. Because there I can really say if I know it early on I can… I have a bigger chance to be cured. That is the difference yes. (Participant 5).

#### Disease factors

The characteristics of the genetic variant were especially important for participants’ decision on disclosure, namely, the risk or probability of actually developing a disease, the severity of the disease, the age in which the disease is most likely to develop, and the availability of alternative ways of diagnosis besides genetic testing.

##### Risk level

The probability of developing a genetic disorder due to a certain genetic variant was relevant results that participants requested in order to make their decision on disclosure. The mutations in the parkin gene represent a very low risk of developing the Parkinson disease while the example of Huntington disease represented a high risk, as a mutation in the Huntingtin gene will always lead to the manifestation of the disease.

Participants who decided for disclosure of a low risk level result stated that they wanted to know any genetic results on their health, as it might be useful in the future.

Yes… I want to know everything, if that is possible. Even if it is not sure, that it will develop, but…yes. (Participant 14).

The main motivation for those wanting to know high risk level results was the desire to be able to plan their future and take the disease into account when making important life decisions. While some considered not working as hard as they do, others would rethink their family planning.

… I want to know it. In general, if I would have… so I can organize my life until it actually breaks out or first symptoms show. […] Yes, so I can still plan my life. Like go on holydays sometime and travel. Otherwise I always postpone it and like this I could go sooner. (Participant 15).

Some participants argued that a high risk combined with the absence of medical interventions would be a motivation against disclosure. They felt a result like this would prevent them from living a full life in the present as constant worries for the future would prevail.

##### Disease severity

Closely connected to risk level is the severity of a disease related to a certain genetic variant. Here, participants differentiated between mild diseases that have a low impact on the utilization of resources, comorbidities, and mortality and severe diseases that have a high impact. Those asking for disclosure for results connected to mild diseases argued that they did not expect to suffer from anxiety upon learning of such results. Instead, they valued the opportunity to inform themselves about the disease and mentally prepare themselves before a potential outbreak.

Yes. You will always have a little bit of fear as well. But you may be able to do something if it breaks out, maybe you can alleviate it. (Participant 17).

Similarly, those asking for disclosure of results for a severe disease evaluated the chance to know their risk of developing a disease that might have a great impact on their life as favorable, because they wanted to be able to plan their life accordingly.

But yes… so you can take provisions and I think the earlier the better. You can also prepare mentally somehow… (Participant 11).

Participants opting against disclosure for mild diseases as well as those deciding against results relating to severe diseases stated that they feared knowing their results would lead to anxiety. While the anxiety caused by variants related to mild diseases was evaluated as unnecessary since the disease will have relatively minor consequences for their lives, participants feared that the worries caused by severe diseases would affect their life choices in a negative way.

That you maybe will already fixate on it too much. Also I think you maybe already feel a tremor even if there is none. That is a shame if you already worry too early about something that probably or maybe will never come. (Participant 5).

##### Age of onset

The age of onset of a disorder related to a genetic variant proved to be an influential factor for participants’ decision. Generally, participants rather wanted to be informed about diseases with an early onset. Illnesses that are estimated to only develop after the age of 60 were considered less relevant. However, participants expected that their preference will change over time and with advancing age.

(…) one should always be able to change one’s mind. […] Because one will maybe be more adult and then one might have a different opinion. (Participant 9).

Some participants above the age of 60 indicated that they did not want to know any genetic results, even if the consequences of a genetic mutation might affect them soon. They argued that genetic results are no longer relevant for them, but they participated mainly for the benefit of their descendants.

(…)Yes for my descendants. Because for myself it is not that important… let’s say it is important, but it does not benefit me anymore. The clinical exams yes, but the research results are for my descendants. (Participant 3).

##### Chances for diagnosis

Another interesting factor that emerged from the interviews was the question concerning alternative possibilities for diagnosis. If obtaining the genetic results from the CHRIS study was the only chance for participants to receive a diagnosis, some wanted to be informed about results that had characteristics that would normally lead them to reject disclosure. The same applied also if getting a diagnosis would be possible but potentially very difficult or require invasive testing.

If there is no other way of identifying the disease, I would like to know. Otherwise not. (Participant 5).

#### Impact on genetic relatives

Another important decision factor was the significance of genetic results for participants’ children and relatives.

Heredity.

The heritability of a genetic defect greatly influenced the decision of participants that already had or planned to have children. Many participants wishing to have children in the future wanted to know their risk, as they did not want to pass on any disease to their children and would consider pursuing alternative family planning options.

Well then I would wonder if I even want to have children, because it is an imposition. The children cannot decide if they want to have the gene. I think it is a little bit selfish if you then just… just make this decision for the children. (Participant 10).

Others, however, did not want that a mere risk to influence such an important life decision. Several participants demanded specifically not to be informed, as it might generate anxiety in their children or because they felt their children had the right to decide themselves if they wanted to know such results once they are grown up.

No. Even if the kids are… (affected). I already have two children so… […]. I do not want to know that, no. Because there you see no chance somehow, that this can’t be or so. (Participant 04).

Affected relatives.

The prospect that other relatives might be affected by a genetic mutation was evaluated as a less decisive factor as the possibility of passing it onto their children. Many participants felt that their relatives had to decide for themselves if they wanted to take genetic examinations and expressed uncertainty as to whether they would share their results with their family. However, certain participants that had experiences with severe diseases in their family reported it would be important to share any results to enable their relatives to take appropriate action.

### Participants’ misconceptions about genetic results in CHRIS

Throughout the interviews, two major misconceptions regarding the return of unsolicited results in the CHRIS study were identified.

Firstly, during the informed consent process of the CHIRS study, participants were informed that researchers are not actively searching for diagnostic variants. This fact was explicitly mentioned in the participant brochure, in the introductory video to the CHRIS study that all participants viewed prior to participating in the study, and in the consent form. It was also stated that in rare cases in which relevant results were identified during research, participants will be contacted according to their preferences indicated on the consent form. Despite this, many participants assumed that the researchers would actively look through their data to identify genetic variants that might lead to the development of a disease and they expected to be contacted in case relevant results were available. This led participants to presume that if the CHRIS study did not contact them, no relevant genetic mutations had been detected during the active screening.

Secondly, it was detected that while people were aware of the possibility to receive genetic results or even expected it, there was limited understanding as to the meaning of genetic results. During the interviews, participants expressed surprise when the three examples of genetic variants and their health implications were presented to them. Participants stated that they were unsure of what genetic results are and that they were unaware of the broad range of possible results in terms of risk, the severity of potential health implication, and treatment possibilities.

The results from the questionnaire demonstrated that participants have an average knowledge of genetics. Participants had to rate 9 statements on inheritance and the development of genetic diseases true or false. In sum, participants answered 105 of 180 questions correctly and 44 incorrectly and 31 questions remained unanswered. Regarding their self-evaluation, 9 participants evaluated their knowledge on genetics as very low and 5 as low and 6 indicated to know a bit. None of the participants self-reported as having a good or very good knowledge. Also, in the questionnaire, the participants expressed the feeling that their level of knowledge is either equal to the general public or lower. The high number of unanswered questions from the questionnaire and the low self-evaluation indicate that participants have a basic knowledge on genetics.

### The opinion and role of general practitioners and the genetic counsellor in ROR

All GPs supported the return of genetic results as being useful and a right of the participants but they also voiced several concerns. They were especially worried that genetic results might create fear and anxiety among their patients. Further, they were concerned that patients would misinterpret genetic results as a clinical diagnosis. They expressed concern on how participants might react, when learning about their elevated risk to develop certain diseases. They argued that as the general population has no experience in receiving and interpreting genetic results, they might be overwhelmed and over-interpret their predispositions. This might lead them to take unnecessary medical interventions. They emphasized that research results must be clinically validated before returning them.

At the moment I think so because the awareness of how to deal with such results is not very widespread yet. I think this will rather have a shocking effect… (General Practitioner 3).

Another concern voiced was that the genetic results about a person or a family being affected by genetic disorder might spread around the villages and cause social stigmatization. Therefore, the GPs stressed the need for utmost discretion when sharing genetic results.

… it is a small country and like I said, news travel fast. And then people are quick to say… oh in this family, they have this and that gen and so on… Honestly, I would be very cautious. Here you can easily stir up a hornet’s nest. (General Practitioner 2).

When confronted with the three examples of genetic results, the GPs had different opinions on what kind of results should be shared. There were particular concerns about genetic variants that only slightly increase the risk of developing a disease, such as the variants described in the example of Parkinson disease. GPs feared that these results might cause unnecessary anxiety among their patients. In general, however, all GPs agreed that the participant should have the right to decide for themselves what kind of results they want to receive.

… The decision has to remain with the proband, with the human. He has to decide. No discussion. […] This is essential. The owner of the genetic data has to decide. Well the patient or the proband has to decide himself. I think we cannot make the decision for him, and we must not. (General Practitioner 1).

Regarding their own role in the returning process, the GPs stated that they did not feel confident to advise their patients on genetic results, as currently genetics plays a minor role in their daily work and they lack the necessary expertise. While the physicians would like to be informed subject to the consent of the participant, about potential genetic results of their patients, they rather see their role in encouraging patients to seek a specialist’s advice or undergo the necessary examination or treatment.

… I am a general practitioner and I have to take care of so many things. If this is getting so detailed, I would refer to the colleagues, the specialists that work in this field. […] Well I see our role in supporting the patients as good as possible and also in encouraging them to take further steps. (General Practitioner 2).

This is in contrast to the views of many participants who reported having a strong personal and trusting relationship with their general practitioners. Some even preferred to be informed about genetic results by their physician instead of a genetic specialist and others demanded that their results would also be sent to their family doctor after a consultation with a specialist.

Rather the general practitioner because you know him better and longer. And also the doctor knows the patient already for a long time. In the hospital it is more anonymous, isn’t it? You are not there often. I would say rather the general practitioner. (Participant 6).

The genetic counselling service in Bolzano supported the return of genetic results to participants and stressed the importance of an autonomous decision on disclosure by the participants that reflects the genetics unit approach to genetic results. They also thought that the cohort could lead to joint projects on several genetic-related disorders that could potentially lead in collaboration with the healthcare system to preventive health projects. For this type of potential development, appropriate understanding and consent of the individuals would be paramount. However, concerns were raised regarding the enormous workload that might arise for their rather small team of only 4 counsellors. The physicians as well as the genetic counselling group stressed that participants cannot be left alone with their results but that a close collaboration between them and the CHRIS study as well as further specialists and psychologists must be built in order to responsibly advise participants.

## Discussion

In this study, we sought to investigate whether we should return results to research participants, and if so, how should such a policy be designed. Our findings echo other studies that have shown that a majority of research participants would like to receive individual genetic results (Bollinger et al. 2012; Facio et al. 2013; Allen et al. 2014; Middleton et al. 2016; Jamal et al. 2017; Yamamoto et al. 2017, 2018). This study also highlights that research participants want to make an independent decision on the disclosure of their genetic results. As the first study that unpacked respondent choices and decision-making based on the four categories presented, it demonstrates that the amount and kind of feedback that participants want to receive are very diverse and based on intrinsic individual motivations that can be part due to the particular genetic condition.

Most research studies and biobanks are hesitant to offer a broad range of genetic results (McGuire et al. 2014). Concerns have been expressed that returning results with uncertain clinical significance or without clear medical actionability could cause distress and psychological harm to participants as they might be unable to correctly interpret results, overestimate their implications, and even undergo unnecessary medical treatments (Lázaro-Muñoz et al. 2017). Dresser has contested this view arguing that there is limited research on this point and thus insufficient evidence to refuse to feedback results due to concerns regarding the potential psychological impact (Dresser 2014). However, to avoid liability, most researchers limit their output to the genetic variants specified in professional guidelines or evaluation mechanisms that have been developed in order to assess the relevance of results for research participants (ACMG 2015; Berg et al. 2013, 2016; Cassa et al. 2012; Matthijs et al. 2016). All these recommendations state clinical validity and medical actionability as the main criteria for returning genetic results (Lázaro-Muñoz et al. 2017) and do not consider participant autonomy and their preferences.

The findings of our study indicate that there is a disconnect between patient preferences and the narrow margin of results researchers deem appropriate for sharing. Researchers and biobanks rely on “clinical utility” (i.e., whether the results can improve a clinical outcome), whereas our participants’ motivations rest more on “personal utility,” echoing the findings of Urban and Schweda (2018). Personal utility refers to non-clinical uses of the results such as feelings of control and planning for the future (Kholer et al. 2017). However, our study indicates that personal utility and clinical utility are very much intertwined and provide empirical evidence for this need for a shift towards reporting results that have personal utility (Thorogood et al. 2019). Personal decisions are very much linked to clinical outcomes and both must be factored in when deciding on feedback of results.

Having identified that our participants have a preference for the return of results, the CHRIS study now needs to determine the process in case of results and importantly who should be responsible for returning to the participant. We need to be mindful that the main motivation for those against feedback was the expectation that knowing certain genetic results will lead to anxiety, reduce their quality of life, and negatively affect their life decisions. The interviewed GPs and the genetic counsellor also expected that sharing genetic results might lead to worries and anxiety among participants, as they have no experience interpreting and dealing with genetic results. The GPs especially feared that without adequate support and counselling, receiving genetic results might cause harm for the participants. Due to their time constraints and limited expertise on genetic results, the GPs see their role as encouraging participant to seek a specialist’s advice. Thus, the responsibility should not fall to GPs but genetic counsellors, who have the necessary knowledge and experience to address participants’ concerns. Tied up with this is the complex question of whether feedback of results should be classed as a responsibility of research institutions or general responsibilities to research participants involving public health. Resources and expertise available will impact the ability to return results, but owing to the strong preferences of participants to return results, researchers must factor this into the design of a study and facilitate the development of a policy on this topic.

Having unpacked the findings in the study, the CHRIS study set up a collaboration with the local healthcare system and the genetic counsellor unit. A joint committee comprising of ethics board members, researchers, and genetic counsellors will meet ad hoc to assess the ROR cases as they arise. We decided to integrate four examples of genetic results into the consent process of the follow-up phase of the CHRIS study: breast cancer, Parkinson disease, Huntington disease, and malignant hyperthermia. Participants will receive information on these four examples through different channels. With their invitation to the second phase of the study, they will receive a brochure explaining the examples and there will be additional information on the website of the CHRIS study. On this website, four videos (in German and Italian) are also available, in which doctors from the local genetic counselling service explain the four examples. When coming to the CHRIS center, participants will have the chance to watch an introductory video, explaining the second phase of the study. In this video, a brief summary of the examples and their main characteristics will be given. After watching this video, all participants will be asked to indicate for each example, whether they want to be informed or not.

There is now a growing consensus that there is a legal and ethical obligation to inform participants about the possibility or not to receive genetic results from a study (Ralefala et al. 2020). Some results that could be life saving (such as the MH) or actionable within existing healthcare pathways (BRAC 1 and 2) if known and available should be returned.

Our study provides an empirically tested procedure to be followed in designing a returns policy. This process can equally apply to biobanks and research projects that are looking to shift from a “no-return” policy to a return of results policy or a mixed situation and those looking to design an ad hoc returns policy at the outset of their study. For those developing such a policy, we offer four recommendations.

First, although CHRIS has adopted a patient-centric approach that requires an assessment of patient preferences at all stages of the research (Pattaro et al. 2015), the starting premise should be *if* participants want results returned and not *how* or *what* results should be returned. Starting from this point ensures that a policy is participant and not researcher led.

Second, when participants indicate a preference to know, researchers have a responsibility to develop a policy at the outset, in line with participant views. The factors affecting participants’ desire for results to be returned should guide the policy development and not the type of results to be returned. Focusing a policy on the type of results fails to consider the other factors behind participant decision to receive results. Thus, the current approach of the ACMG and ESHG is thus too narrow in focus.

Third, participants are likely to have limited knowledge on genetic results. An important aspect in designing a responsible disclosure process for genetic results is to provide easily accessible and understandable information on all the identified factors that influence participants’ choices. Providing different examples for genetic results proved to be extremely helpful in facilitating participants’ comprehension.

Fourth, the development of this policy must also involve local genetic counsellors and representatives of the local healthcare system. This will enable the identification of who best to feedback results in line with the expertise and resources available to be integrated in the healthcare pathway.

Finally, the development and possible implementation of a policy must be budgeted for. Costs associated with the development of a policy include stakeholder engagement and the development of any necessary educational material. Educational material will also need to be provided during the consent process that informs participants about the return of results policy.

The financial implications of a policy will depend upon whether the results will be actively searched for or if only research results found because of specific inquires will be returned. The costs of appropriate genetic counselling services that could be paid out of research funds or covered through the local healthcare system also need to be considered. This important factor points to the need for early discussion and development of a policy.

## Limitations

Reporting numbers in qualitative studies is controversial (Maxwell 2010). While some argue that numbers allow for precise description and thus increase the meaning of key findings, others fear they can (even unconsciously) lead to a false sense of generalizability of the results and imply the picture of a measurable reality (Neale et al. 2014). For this study, it must be stressed that due to the low number of participants, no conclusions about the prevalence of opinions in the CHRIS population can be drawn. The aim of this study was to collect as many different opinions and arguments as possible but not to assess their prevalence.

## Conclusion

This study aimed to inform the update of the policy on the return of genetic results to research participants of the Cooperative Health Research in South Tyrol (CHRIS) study. Through qualitative face-to-face interviews, we investigated the concerns, needs, and wishes of research participants, local general practitioners, and the local genetic counselling service regarding the return of individual genetic research results. Our findings reveal that participants want to make autonomous decisions regarding the return of their genetic results. However, we were able to identify several common criteria influencing research participants’ decision on disclosure, providing easily comprehendible information on these factors is crucial to enable participants to make an informed and autonomous decisions. Moreover, the expectation to suffer from anxiety has been identified as main reason why research participants decline genetic results. This fear needs to be addressed by setting up a responsible communication process with appropriate genetic counselling in place before any results can be shared. Thereby, local general practitioners can only take over a limited, supporting role as they do not have the necessary genetic and psychological expertise.

## Declarations

### Disclaimer

The funders had no role in study design, data collection and analysis, decision to publish, or preparation of the manuscript.
